# RETRACTION: Effect of Skeletonisation and Pedicled Bilateral Internal Mammary Artery Grafting in Coronary Artery Bypass Surgery on Post‐Operative Wound Infection: A Meta‐Analysis

**DOI:** 10.1111/iwj.70351

**Published:** 2025-03-17

## Abstract

**RETRACTION:** NieC. , DengY. , and LuY. , “Effect of Skeletonisation and Pedicled Bilateral Internal Mammary Artery Grafting in Coronary Artery Bypass Surgery on Post‐Operative Wound Infection: A Meta‐Analysis,” International Wound Journal 21, no. 2 (2024): e14424, 10.1111/iwj.14424.PMC1082871737818829

The above article, published online on 11 October 2023, in Wiley Online Library (http://onlinelibrary.wiley.com/), has been retracted by agreement between the journal Editor in Chief, Professor Keith Harding; and John Wiley & Sons Ltd. Following an investigation by the publisher, all parties have concluded that this article was accepted solely on the basis of a compromised peer review process. The editors have therefore decided to retract the article. The authors did not respond to our notice regarding the retraction.

**RETRACTION:** C. Nie , Y. Deng , and Y. Lu , “Effect of Skeletonisation and Pedicled Bilateral Internal Mammary Artery Grafting in Coronary Artery Bypass Surgery on Post‐Operative Wound Infection: A Meta‐Analysis,” International Wound Journal 21, no. 2 (2024): e14424, 10.1111/iwj.14424.PMC1082871737818829

The above article, published online on 11 October 2023, in Wiley Online Library (http://onlinelibrary.wiley.com/), has been retracted by agreement between the journal Editor in Chief, Professor Keith Harding; and John Wiley & Sons Ltd. Following an investigation by the publisher, all parties have concluded that this article was accepted solely on the basis of a compromised peer review process. The editors have therefore decided to retract the article. The authors did not respond to our notice regarding the retraction.

